# Effects of Biofeedback Training on Esophageal Peristalsis in Amyotrophic Lateral Sclerosis Patients with Dysphagia

**DOI:** 10.3390/jcm9072314

**Published:** 2020-07-21

**Authors:** Jerzy Tomik, Klaudia Sowula, Piotr Ceranowicz, Mateusz Dworak, Kamila Stolcman

**Affiliations:** 1 ENT Department, Faculty of Medicine, Jagiellonian University Medical College, Jakubowskiego 2, 30-688 Krakow, Poland; sowula.k@gmail.com (K.S.); dworakmateusz90@gmail.com (M.D.); kamila.stolcman@gmail.com (K.S.); 2 Department of Physiology, Faculty of Medicine, Jagiellonian University Medical College, Grzegórzecka 16, 31-531 Krakow, Poland; piotr.ceranowicz@uj.edu.pl

**Keywords:** biofeedback training, esophageal peristalsis, dysphagia, ALS

## Abstract

Esophageal manometry (EM) could serve as an objective method for the detection of esophageal peristalsis in patients with amyotrophic lateral sclerosis (ALS). In this group of patients, biofeedback training (BT) using the EM procedure is a promising method for the rehabilitation of swallowing function. A total of 20 ALS patients with clinical evidence of dysphagia and who met WFN criteria were recruited for this study. The standard transnasal EM with solid-state transducers was performed, and swallows with water and saliva were initiated in all subjects and repeated at 30-s intervals. The median upper esophageal contractile amplitude, duration, and velocity results during the wet and dry swallows were evaluated and compared in both the ALS and the control groups. In ALS patients, in contrast to the control, significant abnormalities in all EM parameters were recorded, which implies a specific pattern of esophageal peristalsis. Twelve months after BT, the body mass index (BMI) of ALS patients who underwent BT (ALSBT) was compared to the BMI of those who did not (ALS1)—compared to the ALS1 group, ALSBT patients showed a slightly smaller drop in BMI value. We presume that BT using EM can be a promising tool for the improvement of the swallowing mechanism in ALS patients.

## 1. Introduction

Amyotrophic lateral sclerosis (ALS) is a progressive neurodegenerative disease. A major problem in patients suffering from ALS is swallowing dysfunction, which increases the risk of aspiration pneumonia and nutritional status disorders [1,2].

Swallowing disturbances in ALS patients are difficult to assess objectively. Currently, there are only a few techniques available to reliably assess the stages of ALS swallowing dysfunction, which include videofluoroscopy (VF), fiberoptic endoscopic examination of swallowing (FEES), and various clinical scales based on symptomatology (e.g., ALSSS and ALSFRS-R) [2,3,4].

The swallowing mechanism in ALS patients has not been systematically studied, whereas bedside examination and clinical evaluation do not ensure accuracy. Esophageal manometry (EM) is an objective manometric method for esophageal peristalsis failure detection and rehabilitation in ALS patients. In order to improve the swallowing function in ALS patients with symptoms of dysphagia, we used the biofeedback (BF) method for the rehabilitation of the disturbed mechanism of the esophageal phase of swallowing (i.e., the rehabilitation of the upper one-third of the esophageal striated muscles). 

Biofeedback training (BT) using an esophageal manometry (EM) procedure is a promising method for patients who refuse to undergo percutaneous endoscopic gastrostomy (PEG) symptomatic treatment [4,5].

Detection and measurement of the esophageal phase of dysphagia can be of great importance in contributing to a better understanding of the pathophysiology of dysphagia and improving future rehabilitation of swallowing dysfunctions in ALS patients [6]. Although several studies have established PEG in ALS patients as a new and commonly used symptomatic treatment for dysphagia, it has not been the choice of all patients. Therefore, introducing a better swallowing disturbance therapy could improve the nutritional status of patients and help them maintain functions necessary for oral food intake [4,7].

Aim of the study: The aim of this study was to evaluate the effectiveness of BT in the rehabilitation of impaired swallowing mechanisms in ALS patients and to assess its effectiveness in the rehabilitation of disturbed swallowing mechanisms in dysphagic ALS patients.

## 2. Materials and Methods

### 2.1. Subjects

The group of patients originally consisted of 8 (40%) men and 12 (60%) women with clinical evidence of dysphagia. Dysphagia was assessed according to the ALS swallowing severity (ALSSS) scale, which is a 10-point scale comprising a part of the ALS severity (ALSS) scale used to evaluate symptoms of swallowing in ALS patients [8]. These patients had ALSSS scores ranging from 5 to 8 points. Mean age at entry was 56.8 ± 11.4 years (range: 36−74 years). ALS was diagnosed in the outpatient clinic dedicated to ALS patients and affiliated with the Department of Neurology of the Jagiellonian University Medical College in Krakow, Poland. The diagnosis of ALS was established by the same neurologist according to the revised El Escorial World Federation of Neurology criteria [9].

The research was carried out over a period of two years. Initially, the study included a group of 34 patients with swallowing disorders; however, at enrollment for the experiment, 5 people had dropped out, 4 people had died, and the general condition of 5 patients had deteriorated, which in keeping with the underlying exclusion criteria made them not eligible for the study.

The disease duration was calculated from the first bulbar symptoms onset, and it ranged from 5 to 72 months (mean: 16.5 ± 14.9 months). The duration of dysphagia ranged from 1 to 26 months (mean: 8.6 ± 6.4 months) (Table 1).

The control group that qualified for the manometric study consisted of 20 sex- and age-matched healthy volunteers (11 men and 9 women) who declared no swallowing problems and whose mean age was 48.0 ± 12.3 years (range: 35–65 years) (Table 2).

The inclusion criteria for the study group were: (1) bulbar symptoms in the clinical examination, (2) clinical evidence of dysphagia in the swallowing severity scale (a score of ≤7 points (i.e., prolonged time swallowing small bits of food) but >5 point (i.e., liquid diet)), (3) forced vital capacity (FVC) >50% (the obligatory standard spirometry was carried out in the sitting position), and (4) written informed consent.

Criteria excluding patients from the study were as follows:

1. Age below 30 years and above 75 years;
2. Significant deterioration of the swallowing function in the medical history and physical examination (a score of less than 5 points on the ALSSS scale);
  * ALSSS scale—ALS swallowing severity scale (Hillel, 1989)—10 point scale comprising a part of the ALSS scale (ALS severity scale) used to evaluate symptoms of swallowing in ALS patients.
3. Deterioration of the clinical status of a patient during the course of the study (i.e., significant increase in bulbar symptoms, emergence of subjective respiratory dysfunction, cachexia, or intensification of limb symptoms making the patient unable to turn up for the study);
4. FVC <50%, measured obligatorily by spirometry both in the seated and standing positions.
5. Anatomical changes in the oral cavity, pharynx, or esophagus that prevent objective assessment;
6. A swallowing disorder which is not the consequence of the primary disease (e.g., patients after surgical operations on neck, local radiotherapy, or other causes of esophageal peristalsis disturbance);
7. Taking medication which negatively affect the motor functions of the esophagus (e.g., calcium blockers, beta blockers, nitrates);
8. Comorbidities such as cardiovascular disease, especially cardiac arrythmia, as well as other digestive tract disorders;
9. Lack of the patient’s consent to take part in the study.

Clinical assessment of each studied patient was conducted comprehensively by the following means:

- Neurological examination;
- Clinical evaluation of the severity of swallowing disturbance (ALSSS);
- Evaluation of dysphagia according to ALSSS scale;
- Evaluation of clinical condition according to semiquantitative scales such as Norris, ALS Functional Rating Scale Revised (ALSFRS-R) [10];
- Calculation of the body mass index (BMI).

The ALS Functional Rating Scale Revised is an instrument for evaluating the functional status of patients with ALS. It can be used to monitor functional changes over time. It takes into account the following functions: speech, salivation, swallowing, handwriting, cutting food and handling utensils, dressing and hygiene, turning in bed and adjusting bedclothes, walking, climbing stairs, and breathing.

The study protocol was approved by Bioethical Committee of Jagiellonian University in Krakow (KBET/123/B/2004). All subjects provided their informed consent to participate in the study.

### 2.2. Methods

Manometric examination of the upper part of the gastrointestinal tract was performed with the use of a computerized Synectics System (Sweden) consisting of a Polygraph VIII, a solid-state catheter (number P 33-4200 DM60; Küngsberg Instruments, Pasadena, USA), and the software Polygram Upper GI Edition, version 5.06 (Synectics).

The study was performed in the sitting position according to the technique described by Castell and Castell [11]. The flexible esophageal catheter with solid-state transducers was positioned in the esophagus transnasally.

Esophageal pressure values were estimated directly by the solid-state transducers in the catheter. Four manometric transducers were located in the distal part of the catheter, with the first, second, and third, and the third gaps between them measuring 3, 2, and 5 cm in distance, respectively. The proximal part of the esophagus was evaluated by locating the proximal transducer 1 cm below the distal border of the upper esophageal sphincter (UES). The most important observations were collected from the distal probe with circumferential possibility of measuring. 

Each subject performed five dry swallowing maneuvers (saliva only) and five wet swallowing maneuvers (about 10 mL of water per each swallowing act). All patients with dysphagia presented an individual rhythm of successful swallowing, which determined the total examination time.

Before insertion of the catheter, each patient was put in a sitting position in front of the computer screen. After catheter insertion, the patient was given a 5-min rest for habituation and better tolerance of the catheter. During this period, patients observed the manometric changes of their saliva swallowing, coughing, and breathing in real time without the examiner’s assistance.

The following parameters were measured automatically in both ALS and control subjects by the computer system: (1) upper esophageal contractile amplitude, (2) upper esophageal contractile duration, and (3) upper esophageal contractile velocity during the wet and dry swallows.

Patients who agreed to the protocol of esophageal rehabilitation underwent a cycle of five sessions of BT using EM. The same EM procedure as described previously was used. 

Firstly, swallows with 5 to 10 mL of water (wet swallows) and saliva (dry swallows) were initiated in each subject and repeated at 30-s intervals. After initial examination, the procedure was repeated in 10 out of 20 ALS patients, 10 times each, at 5-day intervals with solid and liquid meals, and the procedure was interrupted by Valsalva and Mueller maneuvers with profound intraesophageal pressure changes. Patients observed their frequency of primary, secondary, and nonperistaltic waves and learned about their parameters of swallowing function. Twelve months after BT, the body mass index (BMI) of the ALS patients who underwent BT (ALSBT) was compared with the BMI of the ALS patients who did not (ALS1).

The effectiveness of swallowing disturbance rehabilitation in ALS patients with dysphagia was evaluated. The following parameters were measured: resting intraesophageal pressure at the end of exhalation and mean amplitude, duration, and velocity during dry and wet swallowing. Primary and secondary peristaltic waves and presence of nonperistaltic waves were also assessed in ALS patients with dysphagia. The parameters were estimated in patients twice: once before BT therapy and once 12 months after it.

The rehabilitation EM procedure was performed according to the following steps:

1. The measurement of maximal frequency of successful primary wet and dry swallows;
2. The measurement of maximal frequency of successful primary swallows during the solid meal (the central part of bread without crust, with butter);
3. The measurement of maximal frequency of successful primary swallows during liquid meals (blended juice).

During swallows (steps 2 and 3), patients were requested to thoroughly prepare the given bit orally before swallowing. Patients chose the most convenient of four head positions for successful swallowing, i.e., left turn position, right turn position, flexion of the neck, or extension of the neck. Head positions which aggravated dysphagia were excluded.

### 2.3. Statistical Analysis

Qualitative data are presented as counts and percentages. Quantitative data are presented as means (along with 95% confidence intervals) and SDs. Chi-squared tests, Student’s *t*-tests for independent samples, nonparametric Mann−Whitney−Wilcoxon tests, and Pearson’s correlation tests were conducted in STATISTICA v.8.0. A *p* value ≤0.05 was accepted as statistically significant.

### 2.4. Sample Size Analysis and Power Estimation

It was estimated that with 20 patients per group at the two-sided 5% significance level the study had the following:

- A 99% power to detect the difference of 30 in amplitude of a wet swallow, assuming mean 60 and SD 20 in the control group;
- A 99% power to detect the difference of 1 in duration of a wet swallow, assuming mean 2.5 and SD 0.7 in the control group;
- An 89% power to detect the difference of 0.5 in velocity of a wet swallow, assuming mean 2.5 and SD 0.5 in the control group;
- A 100% power to detect the difference of 40 in amplitude of a dry swallow, assuming mean 40 and SD 25 in the control group;
- An 89% power to detect the difference of 2 in duration of a dry swallow, assuming mean 2.5 and SD 2 in the control group;
- An 89% power to detect the difference of 0.5 in velocity of a dry swallow, assuming mean 4 and SD 0.5 in the control group.

## 3. Results

The mean value of the Norris scale scores was 72.7 ± 27.2 points (range: 46−101 points; 95%CI = 60.8−84.6). The mean value of the ALSFRS scores was 22.9 ± 8.5 points (range: 16−37 points; 95%CI = 19.2−26.6). The mean value of the clinical scale of swallowing scores was 6.2 ± 1.6 points (range: 5−7 points; 95%CI = 5.5−6.9).

**Figure 1:**
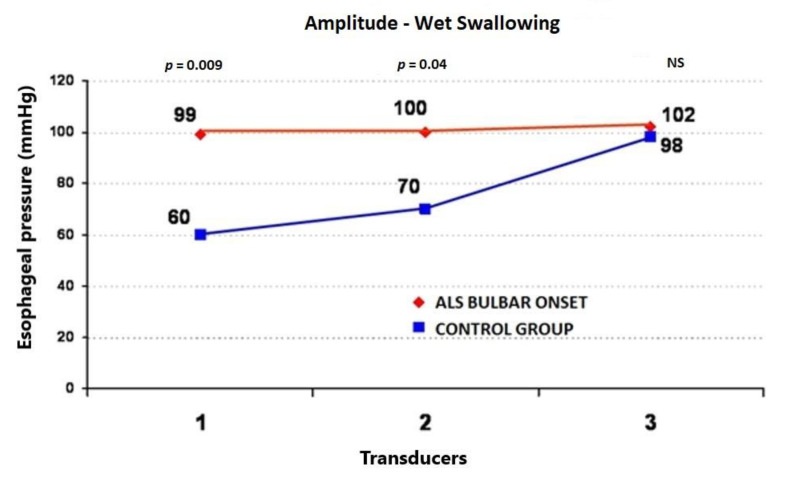
Comparison of the mean values of peristaltic wave amplitude during wet swallows between ALS patients with dysphagia and the control group (from three transducers).

**Figure 2:**
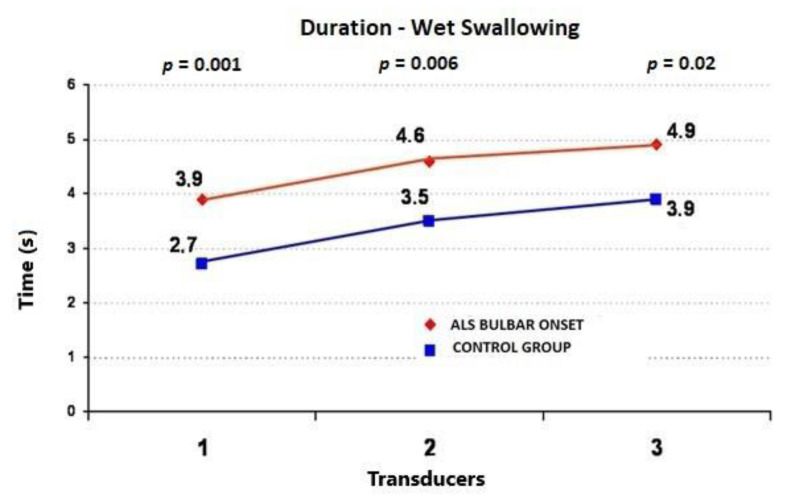
Comparison of mean values of peristaltic wave duration during wet swallows between ALS patients with dysphagia and the control group (from three transducers).

**Figure 3:**
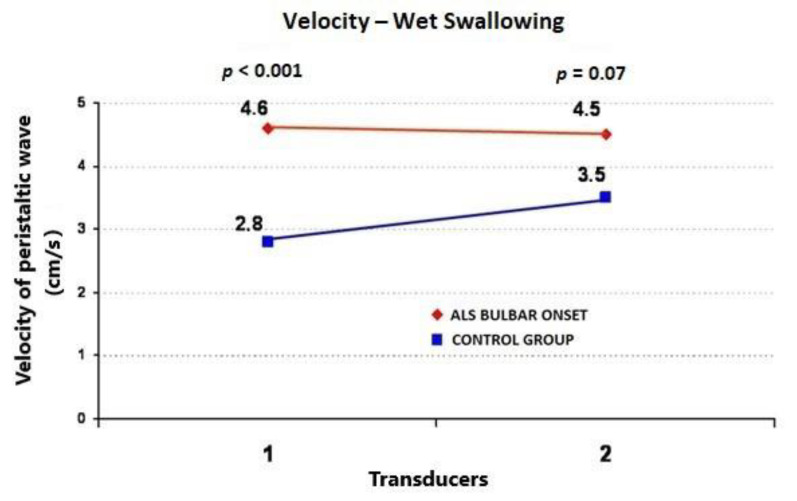
Comparison of mean values of velocity of peristaltic wave propagation during wet swallows between ALS patients with dysphagia and the control group (from two transducers).

**Figure 4:**
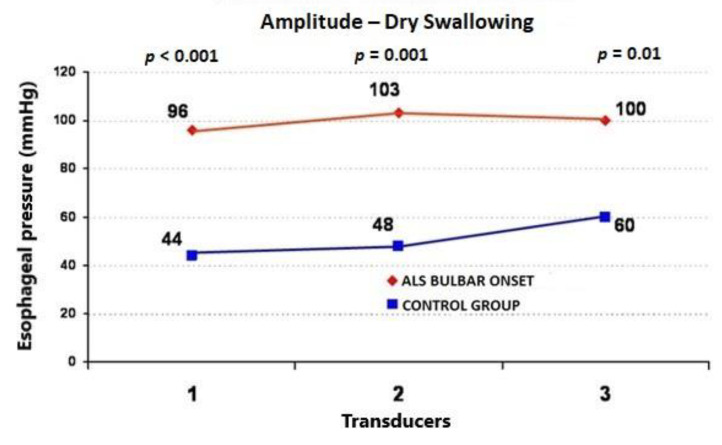
Comparison of mean values of peristaltic wave amplitude during dry swallows between ALS patients with dysphagia and the control group (from three transducers).

**Figure 5:**
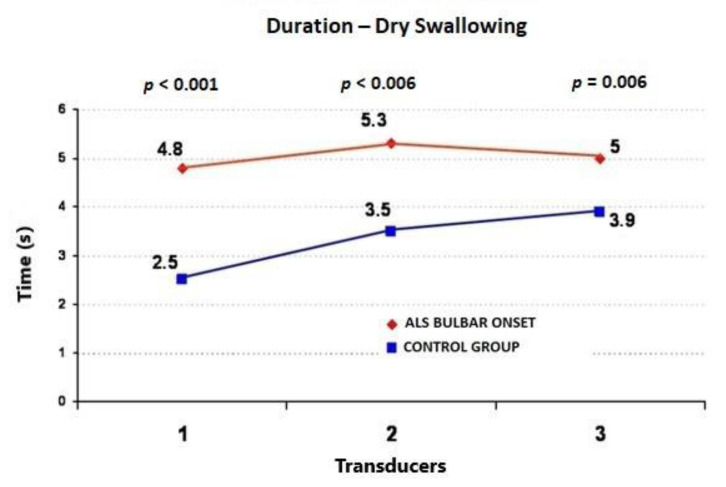
Comparison of mean values of peristaltic wave duration during dry swallows between ALS patients with dysphagia and the control group (from three transducers).

**Figure 6:**
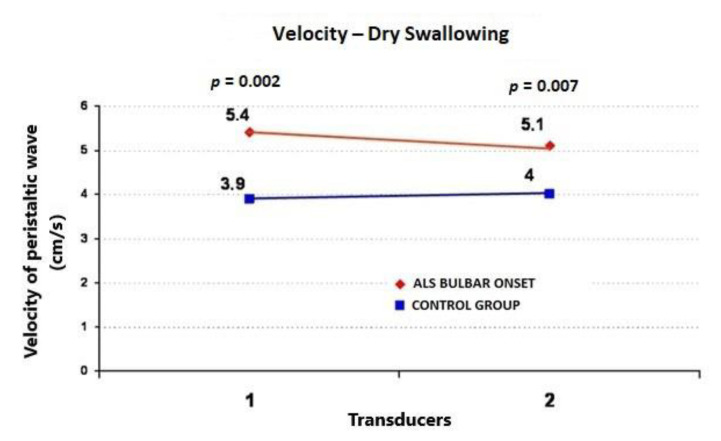
Comparison of mean values of velocity peristaltic wave propagation during dry swallows between ALS patients with dysphagia and the control group (from two transducers).

During the wet swallows, the median upper esophageal contractile amplitude values for ALS vs. control subjects were 99 ± 58 (95%CI = 73.6−124.4) vs. 60 ± 22 mmHg (95%CI = 50.4−69.6) (*p* = 0.009), respectively; the median upper esophageal contractile duration values for ALS vs. control subjects were 3.91 ± 1.4 (95%CI = 3.3−4.5) vs. 2.7 ± 0.7 s (95%CI = 2.4−3.0) (*p* = 0.001), respectively; and the median upper esophageal contractile velocity values for ALS vs. control subjects were 4.6 ± 1.8 (95%CI = 3.8−5.4) vs. 2.8 ± 0.4 cm/s (95%CI = 2.6−3.0) (*p* < 0.001), respectively (Figure 1, Figure 2 and Figure 3). During the dry swallows, the median upper esophageal contractile amplitude values for ALS vs. control subjects were 96 ± 59 (95%CI = 70.1−121.9) vs. 44 ± 25 mmHg (95%CI = 33.0−55.0) (*p* < 0.001), respectively; the median upper esophageal contractile duration values for ALS vs. control subjects were 4.8 ± 1.9 (95%CI = 4.0−5.6) vs. 2.5 ± 1.7 s (95%CI = 1.8−3.2) (*p* < 0.001), respectively; and the median upper esophageal contractile velocity values for ALS vs. control subjects were 5.4 ± 2.0 (95%CI = 4.5−6.3) vs. 3.9 ± 0.5 cm/s (95%CI = 3.7−4.1) (*p* = 0.002), respectively (Figure 4, Figure 5 and Figure 6). 

Twelve months after BT, the frequency of primary peristaltic waves in ALSBT patients increased from 1.8 ± 0.4 (95%CI = 1.6–2.0) to 7.0 ± 1.4 per minute (95%CI = 6.1−7.9), with a significant reduction of nonperistaltic waves (from 7.0 ± 1.1 (95%CI = 6.3–7.7) to 4.9 ± 0.3 per ten minutes (95%CI = 4.7–5.1)) and stable pressure values. In ALS1 patients, no changes were observed in previously presented parameters; however, both an increase in the number of nonpropulsive waves and a decrease in the frequency of primary waves from 1.8 to 0.8 per minute were reported.

**Figure 7:**
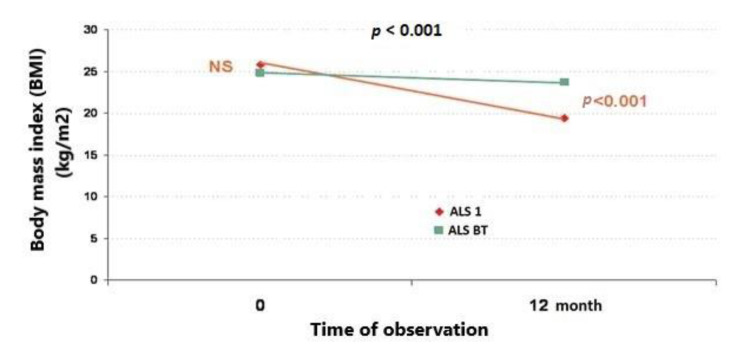
Comparison of BMI between the group of ALS patients who underwent biofeedback training (ALSBT) and the group of ALS patients who did not (ALS1) after 12 months’ observation.

The ALSBT patients showed a smaller decrease in BMI (from 24.8 ± 3.2 (95%CI = 22.8−26.8) to 23.7 ± 3.8 (95%CI = 21.3–26.1)) (*p* = 0.5) compared to ALS1 patients (from 25.8 ± 1.7 (95%CI = 24.7–26.9) to 19.4 ± 2.0 (95%CI = 18.2−20.6)) (*p* < 0.001) during the 12-month period (Figure 7).

## 4. Discussion

The most important therapeutic goal in the case of ALS patients with dysphagia is maintaining adequate nourishment. Sustaining oral alimentation for as long as possible is crucial not only for patients’ quality of life but also for rehabilitation strategies used. 

Among therapeutic approaches supporting swallowing, we distinguish direct methods, which aim to restore stomatognathic and laryngeal movement activity, and indirect methods adopting compensatory strategies. Direct methods include pummeling; stretching exercises; thermal stimulation; and tongue, lower jaw, mouth, palate, and larynx exercises [12]. Indirect mechanisms include various manipulations connected with food intake, such as epiglottic movement swallowing, chin-tuck maneuver (when the tongue base is weakened) [13], Mendelsohn’s maneuver (will-dependent and prolonged lift of hyoid bone–larynx complex and simultaneous stretch of upper esophageal sphincter muscles), head and neck positioning changes, and food consistency modifications (e.g., thickening of foods) [14,15]. Assigning an adequate therapeutic method should be individualized in ALS patients with dysphagia.

The BF technique provides patients with feedback regarding their physiological state changes so that they can learn to consciously modify functions that cannot otherwise be consciously controlled. Information is provided through visualization, acoustic impulses, or with the use of electromyography (EMG) or electroencephalography (EEG). The BF method can be used for the alteration of any physiological response controlled by the central nervous system (CNS), including the excretory function and gastrointestinal smooth muscle response [5]. However, the active and conscious participation of a well-motivated patient is necessary for successful BF training.

In the present study, the detection of pathological changes in the esophageal body coinciding with a relatively well-preserved UES function allowed for conducting BF therapy with the use of visualization and Valsalva and Mueller maneuvers. It was assumed that intramural esophageal neurons were not completely damaged, which was confirmed by direct observation of esophagus activity after performing the Valsalva and Mueller maneuver. Following the Valsalva test, intraesophageal pressure repeatedly increased, inducing secondary peristalsis of the esophageal body.

The analysis of manometric parameters in ALS patients with dysphagia before using the BF method and after a period of 12 months showed a smaller decrease in body mass, an increase in primary peristaltic wave frequency, a reduction in the number of nonperistaltic waves, and a stabilization of pressure values. A larger decrease in body mass, an increase in nonpropulsive waves, and a significant decrease of primary swallowing were observed in patients who did not undergo the BF training. 

The above results indicate that the BF method and EM are effective tools in the therapy of ALS patients with dysphagia, as they stabilize the body mass and prevent the progression of malnutrition and cachexia. Moreover, the method is safe and was well-tolerated by the patients. It is performed at first in the outpatient clinic in the presence of a medical specialist and is later conducted by the patient in the setting of their home.

The framework of behavioral therapy, BF included, is established on three categories of neuronal changes: alteration of synaptic function, structural adaptation, and alteration (or “makeover”) of neuronal nets [16]. Although the effectiveness of BF in patients with dysphagia is frequently emphasized, its exact neurobiological mechanism has not been clear [5].

In the literature, there are no data regarding the rehabilitation of disturbed swallowing mechanisms in ALS with the use of BF and EM. Leder et al. [17] used FEES in order to assess esophageal phase of swallowing dysfunction in ALS patients. FEES is a noninvasive technique that uses visual biofeedback by allowing the patient to track the passage of the bolus, any accumulated food bits, or the adduction and abduction of the vocal cords. We suggest an individual approach for each patient and the adoption of an adequate dysphagia therapy based on FEES findings (e.g., swallowing smaller bits of food, food consistency modification, head positioning changes), with an option to perform multiple fiberoptic endoscopy tests and BF training sessions. In the study mentioned above, however, BF training was not used for the rehabilitation of disturbed swallowing mechanisms.

Manometric methods and BF are used mainly in proctological practice in order to control the function of the external anal sphincter. EMG feedback can be used as an alternative to manometry for controlling the contraction of the external anal sphincter and the inner pelvic floor muscles [5]. BF training is an effective therapy for fecal incontinence with external anal sphincter dysfunction of neuromuscular origin, impaired rectal sensation, or dyssynergia of pelvic floor muscles. 

In the literature, data can be found on the effective use of BF together with surface EMG (sEMG) in the therapy of esophageal phase dysphagia in neurological disorders such as brain stem infarction or head and neck cancers [18]. When using BF together with sEMG, there was an improvement in oral food intake for 92% (39 out of 45) of patients with brain stem infarction and 80% of patients with head or neck cancer [18,19]. A functional improvement of swallowing was also observed in patients with chronic neurogenic dysphagia which occurred as a result of brain stem infarction (in the case of a study conducted on six people) [20,21] or brain stem damage (a study on 10 patients) after the use of BF together with sEMG [5].

## 5. Conclusions

We found that esophageal peristalsis differed between ALS patients and the control group. Our results indicate that BF is a promising method that can be used in an attempt to rehabilitate the disturbed esophageal phase swallowing mechanism in ALS patients.

## Figures and Tables

**Table 1:** Characteristics of the studied groups.

Groups	Age	Sex	WFN * Criteria	Duration of the Disease (Months)	Duration of Dysphagia (Months)
**ALS subjects with dysphagia**	36–74 56.8 ± 11.4 (95%CI = 51.8–61.8)	Females *n* = 12 Males *n* = 8	Definite ALS n = 7 (35%) Probable ALS n = 13 (65%)	5–72 16.5 ± 14.9 (95%CI = 10.0–23.0)	1–26 8.6 ± 6.4 (95%CI = 5.8–11.4)
**Control subjects**	35–65 48 ± 12.3 (95%CI = 42.6–53.4)	Females *n* = 9 Males *n* = 11			

* WFN criteria—criteria for the diagnosis of ALS from the El Escorial World Federation of Neurology.

**Table 2:** The comparison of sex and age between amyotrophic lateral sclerosis (ALS) and control subjects.

Group	Sex		Age (Years)
	Female	Male	x ± SD
**ALS with dysphagia**	12	8	56.8 ± 11.4 (95%CI = 51.8–61.8)
**Control group**	9	11	48.0 ± 12.3 (95%CI = 42.6–53.4)
**Statistical significance, *p* value**	0.343		0.042
